# Annotations

**Published:** 1895-01-19

**Authors:** 


### ANNOTATIONS.

### The Oyster Scare.

THE old question has been revived as to the possibility of receiving the infection of typhoid fever by the consumption of oysters. This is by no means the first time this doubt has been raised, but it may be admitted that on the present occasion it is well supported both by an article by Sir William Broadbent and by a report, taken from the *New York Medical Record,* of a limited outbreak of typhoid which occurred two or three months ago at the Wesleyan University, Middletown, Connecticut, a report which certainly is *ben trovato*, even if to everyone it may not seem entirely convincing. In considering the possibility of the suggested mode of dissemination of typhoid infection it must be confessed that a considerable amount of doubt hangs over the whole question, but whatever further proof may be forthcoming that oysters are capable of conveying typhoid fever, we may feel pretty sure that they do not do so in consequence of a general infection of the great oyster beds by means of London sewage mud. We believe it is true that typhoid fever is at present somewhat more prevalent in London than is usual at this time of year, but when we bear in mind how slight is the usual mortality from this disease, not more than 20 deaths a week in the whole of London in the quarter of the year when it is most prevalent, and remember the enormous consumption of oysters, and when we consider also the high rate of virulence required to explain Sir W. Broadbent's cases on the hypothesis of oyster infection, we may feel perfectly positive that it is not by any general infection of the oyster supply that the mischief has been brought about. In the cases referred to, a husband and wife ate oysters once and both were attacked; two friends had oysters together twice and both were attacked; two young men had an oyster supper, and again both were attacked. An infection so virulent as to produce effects like this ought, if it were wide-spread, to have already caused an equally wide-spread catastrophe, whereas no epidemic at all proportionate has shown itself. We are constrained, therefore, to believe that if the disease has really been conveyed by the oysters, they must have received their infection during the later processes of their manipulation, and not when they were on the beds in the open water, *i.e.*, it must have been a local rather than a general infection. This, of course, is always a possibility, just as the best country milk may have its purity endangered in the course of its retail distribution. It is to the final fattening, and to the methods of distribution, that attention must first be directed in the efforts which will doubtless be made to maintain the reputation of this succulent bivalve.

### Cycling and Heart Disease.

IN addressing the London Medical Society on "Cycling and Heart Disease," Sir Benjamin Richardson was peculiarly happy, both topics being subjects to which he is known to have paid much attention, and it was interesting to observe that the enthusiastic cycler when brought face to face with the question of the influence of cycling on the heart spoke with very considerable caution. Not that he hesitates in the slightest concerning the advantages of that form of exercise, nor even as to its utility to certain people actually suffering from heart disease; of these things he seems assured, but he is also clear that when carried to extreme, injury results—an injury which falls primarily upon the heart. The effect of cycling upon the heart is peculiar, and, according to Sir Benjamin Richardson, different from what is found in other forms of exercise, except running. The pulse is always quickened, becomes full and bounding; the heart is for the time somewhat distended; it contracts forcibly, and its second sound is often accentuated. Practice makes no difference to this; even the most regular riders have their pulses quickened by the exercise. Attention was drawn to the curious similarity between the effect upon the heart of the two very different stimulants—cycling and alcohol. The active heart, the bounding pulse, the free supply of blood to the tissues are for the time curiously alike, with this difference, however, that with alcohol they are of but short duration, and more has to be taken, whereas in cycling the condition can be maintained. Still, cycling is a heart stimulant, and it is an important question to decide how far such repeated stimulation can be safely indulged in. Sir Benjamin Richardson was sure that the immediate risk of cycling so far as the heart is concerned is very small. He had never, during seventeen years of observation of riders in the field, seen anyone have to dismount in consequence of cardiac distress or embarrassment; but as regards its after effect when indulged in to any considerable extent, he spoke with caution, saying that there had hardly yet been time to determine what the ultimate effect might be. He was, however, quite sure that many accomplished and skilful riders had ultimately succumbed prematurely to disease of the heart and vascular system. Caution, then, was necessary, but this applied to what might be called excessive, rather than ordinary riding. Curiously enough it would seem that harm from cycling is more likely to result to young people than to the more advanced in life, chiefly, however, from the tendency of the young to over-do it. When older people find that cycling makes them uncomfortable they give it up; young people, however, put all their soul into it, and the over stimulation of the heart during the period of growth produces an over development of heart muscle which more than meets the wants of the economy and which tends soon to degenerate, and in view of the large use of cycles by tradesmen's messengers it is likely that before long we shall recognise this condition of heart as one of the "diseases of occupation." There are some morbid conditions in which cycling has appeared to Sir Benjamin Richardson to be of definite advantage, such as varicose veins, some cases of commencing fatty degeneration of the heart arising from sedentary pursuits, some cases of marked valvular disease, of intermittency and palpitation, and most markedly cases of anæmia in young women. Excessive cycling is another matter, and the warning was given that the tremendous feats of endurance which are undertaken by some athletes cannot fail to be productive of injury. The very best men may not appear to suffer, although even they do not remain at their best for long; but those who are not the very best fail, and they fail by way of the heart and circulation, degeneration taking the place of the hypertrophy which had been produced. It must never be forgotten that in estimating the fitness of a given case for cycling, especially in elderly people, it is as important or even more so, to attend to the condition of the peripheral circulation as to that of the heart itself.

